# Supplementary material for: The global phylogeography of rapidly expanding multidrug resistant Ural lineage 4.2 Mycobacterium tuberculosis
Source: Nat Commun. 2026 Mar 31;17:4654. doi: 10.1038/s41467-026-71193-6 (PMC13201588; doi:10.1038/s41467-026-71193-6)
Supplement: Supplementary file 2 — Supplementary Information [file 41467_2026_71193_MOESM2_ESM.pdf]

Supplemental Materials

Supplemental Data 1: Run accession, country of origin, and estimated sampling date for each sequence in Ural lineage 4.2.1.2.

Supplemental Figure 1: Inclusion criteria for sequences used in this study

Supplemental Figure 2: Maximum-likelihood of phylogeny with all 5909 isolates included in the study

Supplemental Figure 3: Ancestral state reconstruction

Supplemental Figure 4: Time-calibrated phylogeny with comparison clades

Supplemental Figure 5: SAASI sensitivity analysis

Supplementary Table 1: Lineage-defining SNPs for lineage 4.2.1.2

Supplemental Figure 1: Inclusion criteria for sequences used in this study

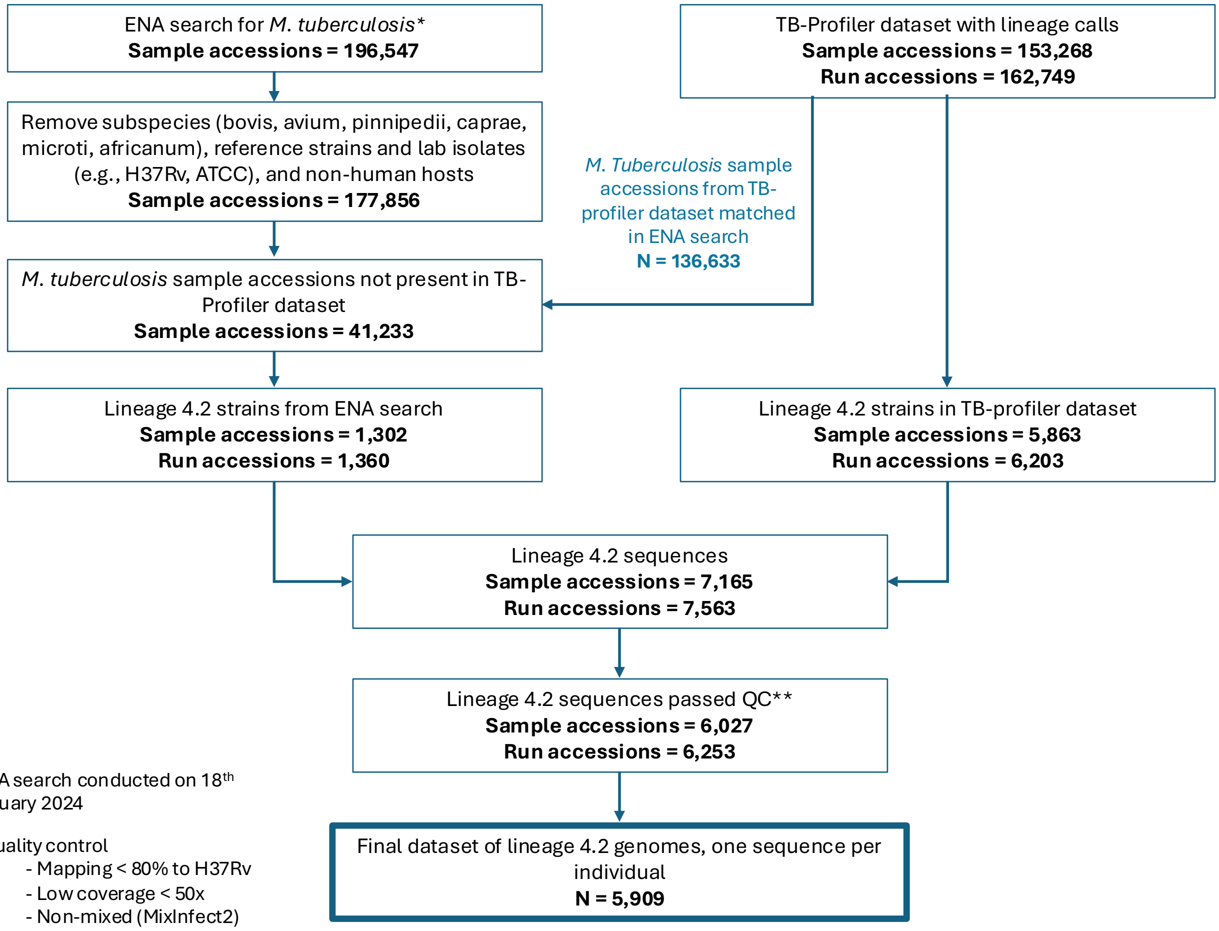

* ENA search conducted on 18th February 2024

** Quality control
- Mapping < 80% to H37Rv
- Low coverage < 50x
- Non-mixed (MixInfect2)

Supplemental Figure 2: Maximum-likelihood of phylogeny with all 5909 isolates included in the study. Strains belonging to lineage 4.2.1.2 are colored in blue; all other strains are colored in grey.

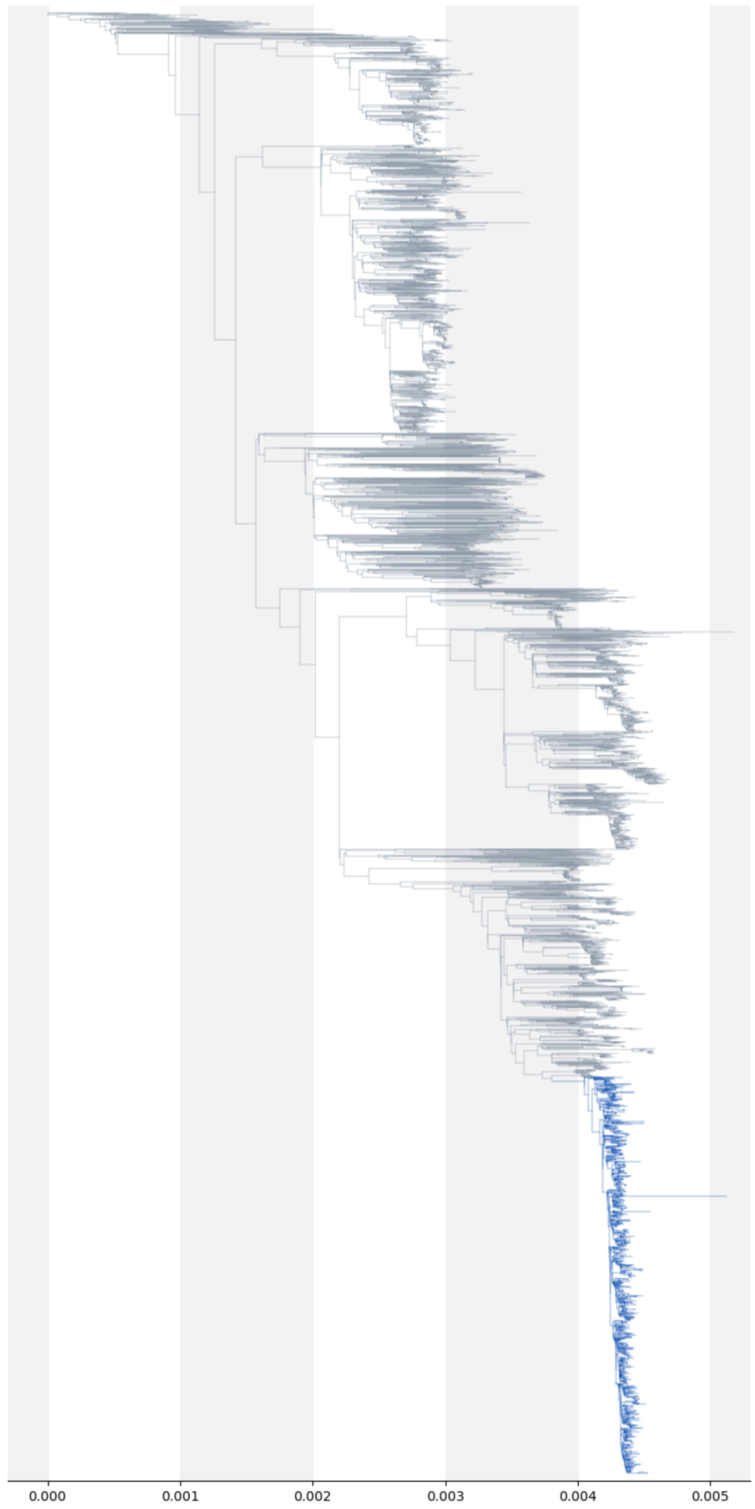

Supplemental Figure 3: Ancestral state reconstruction. (A) Result from SAASI model, including the inferred state of every node pair. (B) Result from ACE model, including the inferred state of every node pair.

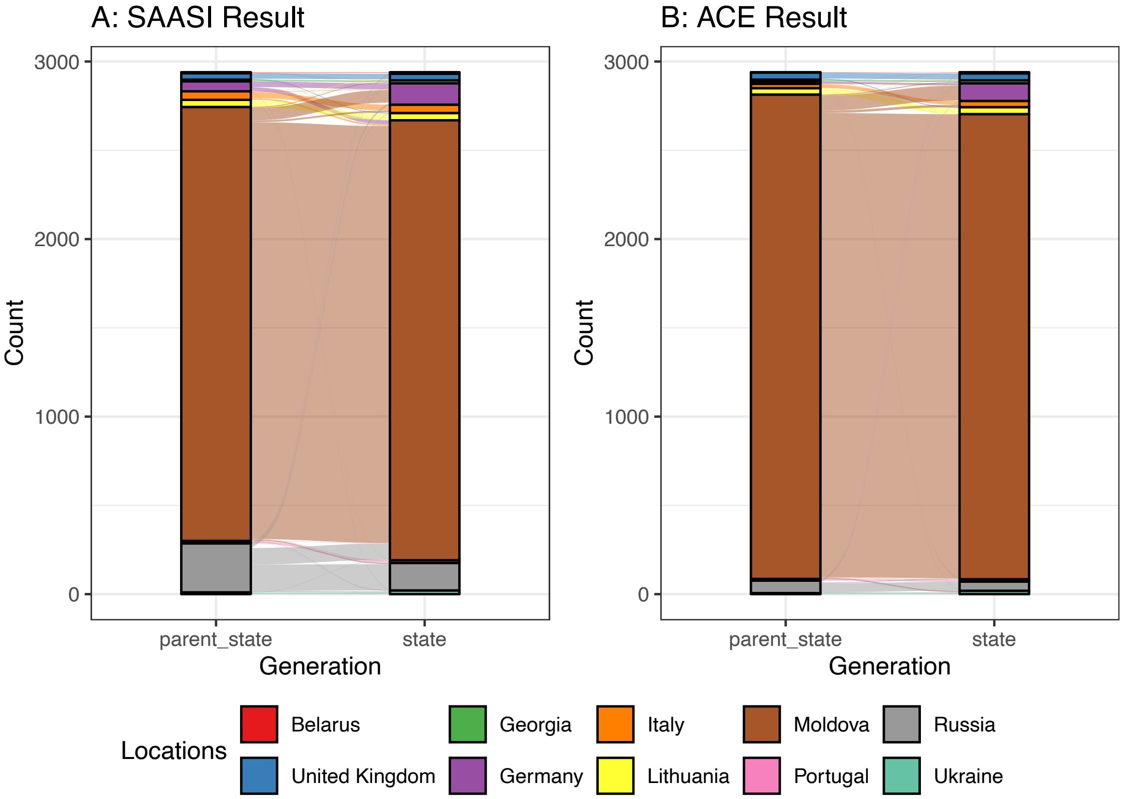

Supplemental Figure 4: Time-calibrated phylogeny with comparison clades

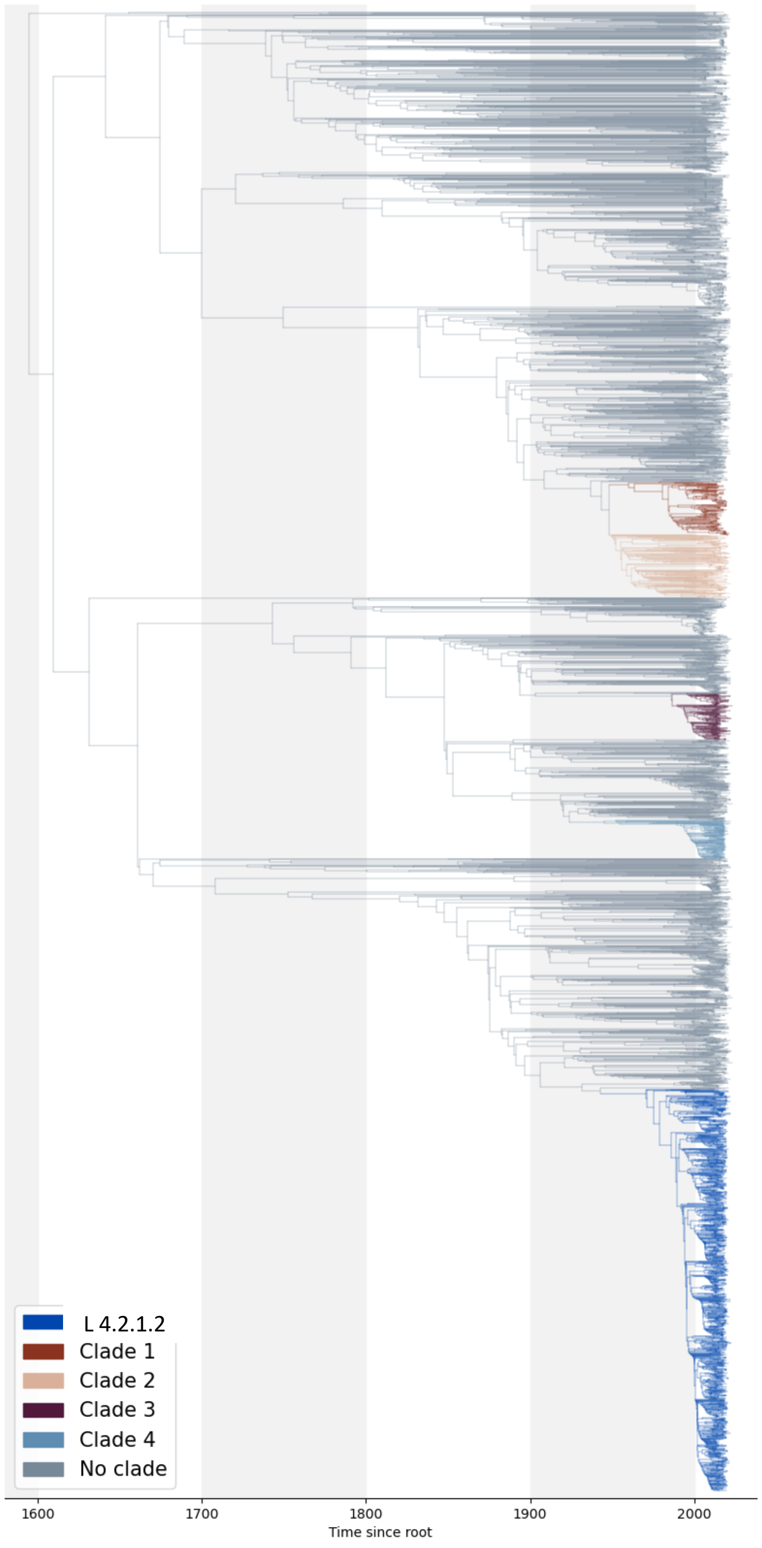

Supplemental Figure 5: SAASI sensitivity analysis

Alluvial plot showing the inferred origin and destination for each migration event, assuming 5% sequencing coverage when there were no data to support sequencing coverage estimates. Sequences from countries with fewer than five isolates have been excluded. “Eastern Europe, Western Asia” includes Belarus, Georgia, and Ukraine; “Norther, Western, Southern Europe” includes Germany, Italy, Lithuania, Portugal, and the United Kingdom. Migration events between countries within these groups have been included in the plot.

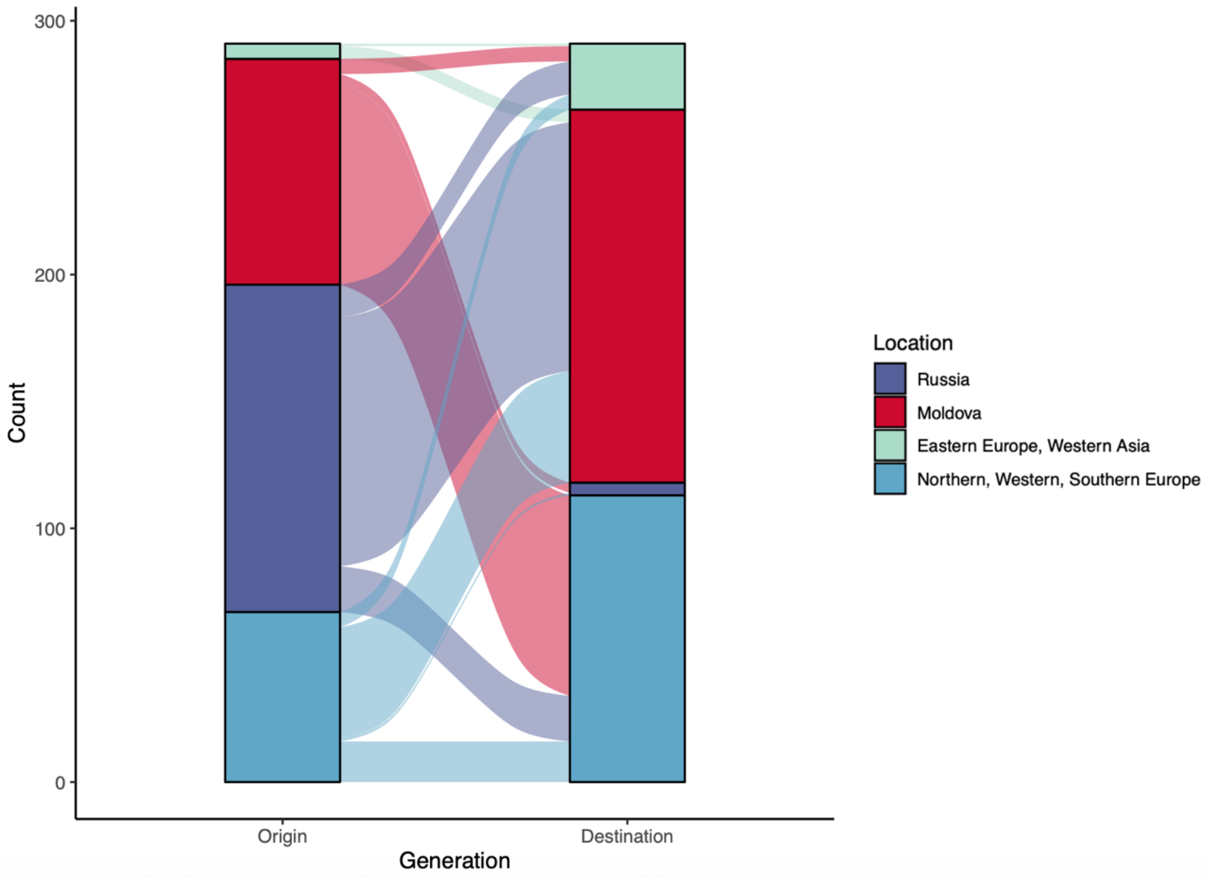

Supplementary Table 1: Proposed lineage-defining synonymous SNPs for lineage 4.2.1.2

H37Rv position	Gene	Locus_Tag	Type	Reference NT	Alternative NT	SNP type	Codon number
69285	.	Rv0064	CDS	G	A	Synonymous	222
240840	mmpL11	Rv0202c	CDS	A	G	Synonymous	151
294938	fadE5	Rv0244c	CDS	C	T	Synonymous	232
306737	cobQ1	Rv0255c	CDS	G	A	Synonymous	374
322677	narU	Rv0267	CDS	C	T	Synonymous	449
491247	fgd1	Rv0407	CDS	C	T	Synonymous	155
608512	.	Rv0516c	CDS	T	G	Synonymous	8
902957	purF	Rv0808	CDS	C	T	Synonymous	283
959936	ercc3	Rv0861c	CDS	C	T	Synonymous	72
962268	.	Rv0862c	CDS	C	T	Synonymous	115
1347296	.	Rv1204c	CDS	C	T	Synonymous	443
1449710	thrA	Rv1294	CDS	C	G	Synonymous	112
1450151	thrA	Rv1294	CDS	C	G	Synonymous	259
1593229	lprH	Rv1418	CDS	C	T	Synonymous	197
1718956	.	Rv1524	CDS	C	T	Synonymous	77
1803371	hisA	Rv1603	CDS	C	G	Synonymous	26
1896311	.	Rv1670	CDS	G	A	Synonymous	64
1926076	.	Rv1700	CDS	C	T	Synonymous	165
2335157	.	Rv2078	CDS	C	G	Synonymous	33
2338768	.	Rv2082	CDS	G	T	Synonymous	20
2463437	.	Rv2199c	CDS	G	A	Synonymous	72
2625924	esxO	Rv2346c	CDS	T	C	Synonymous	83
2626490	esxP	Rv2347c	CDS	G	A	Synonymous	10
2813575	.	Rv2499c	CDS	G	A	Synonymous	52
3298221	fadD22	Rv2948c	CDS	G	A	Synonymous	578
3623222	lpqB	Rv3244c	CDS	T	C	Synonymous	563
3999740	.	Rv3559c	CDS	G	A	Synonymous	232
4003778	fadE31	Rv3562	CDS	C	A	Synonymous	206
4094041	PE_PGRS61	Rv3653	CDS	T	C	Synonymous	34
