# Supplementary material for: The global phylogeography of rapidly expanding multidrug resistant Ural lineage 4.2 Mycobacterium tuberculosis
Source: Nat Commun. 2026 Mar 31;17:4654. doi: 10.1038/s41467-026-71193-6 (PMC13201588; doi:10.1038/s41467-026-71193-6)
Supplement: Supplementary file 3 — Description of Additional Supplementary Files [file 41467_2026_71193_MOESM3_ESM.pdf]

Description of Additional Supplementary Files

File Name: Supplementary Data 1
Description: Run accession numbers, country of origin, and inferred sampling dates for all sequences included in the analysis.
