# Supplementary material for: The global phylogeography of rapidly expanding multidrug resistant Ural lineage 4.2 Mycobacterium tuberculosis
Source: Nat Commun. 2026 Mar 31;17:4654. doi: 10.1038/s41467-026-71193-6 (PMC13201588; doi:10.1038/s41467-026-71193-6)
Supplement: Supplementary file 5 — Reporting Summary [file 41467_2026_71193_MOESM5_ESM.pdf]

nature portfolio

Corresponding author(s): Melanie H. Chitwood, Ted Cohen

Last updated by author(s): 2026-25-01

Reporting Summary

Nature Portfolio wishes to improve the reproducibility of the work that we publish. This form provides structure for consistency and transparency in reporting. For further information on Nature Portfolio policies, see our Editorial Policies and the Editorial Policy Checklist.

Statistics

For all statistical analyses, confirm that the following items are present in the figure legend, table legend, main text, or Methods section.

n/a	Confirmed	
☐	☒	The exact sample size (*n*) for each experimental group/condition, given as a discrete number and unit of measurement
☐	☒	A statement on whether measurements were taken from distinct samples or whether the same sample was measured repeatedly
☐	☒	The statistical test(s) used AND whether they are one- or two-sided<br>*Only common tests should be described solely by name; describe more complex techniques in the Methods section.*
☐	☒	A description of all covariates tested
☐	☒	A description of any assumptions or corrections, such as tests of normality and adjustment for multiple comparisons
☐	☒	A full description of the statistical parameters including central tendency (e.g. means) or other basic estimates (e.g. regression coefficient) AND variation (e.g. standard deviation) or associated estimates of uncertainty (e.g. confidence intervals)
☐	☒	For null hypothesis testing, the test statistic (e.g. *F*, *t*, *r*) with confidence intervals, effect sizes, degrees of freedom and *P* value noted<br>*Give P values as exact values whenever suitable.*
☐	☒	For Bayesian analysis, information on the choice of priors and Markov chain Monte Carlo settings
☒	☐	For hierarchical and complex designs, identification of the appropriate level for tests and full reporting of outcomes
☒	☐	Estimates of effect sizes (e.g. Cohen's *d*, Pearson's *r*), indicating how they were calculated

Our web collection on statistics for biologists contains articles on many of the points above.

Software and code

Policy information about availability of computer code

Data collection	Code used to identify and download for lineage 4.2 M. tuberculosis strains in the European Nucleotide Archive (ENA) is available here: https://github.com/bensobkowiak/ENA_search
Data analysis	All data analysis was performed publicly available software. Sequencing data files were aligned to the H37Rv reference strain (NC_000962.3) using BWA-MEM for both paired and single end read data. Binary alignment (BAM) files were indexed and sorted with SAMtools. Evidence of mixed infection was detected using MixInfect2. Variant calling was conducted using GATK 'HaplotypeCaller' and 'GenetypeGVCFs'. We performed maximum-likelihood phylogenetic reconstruction using IQ-TREE 2. Additional analyses were performed in R, using the packages BactDating, treestructure, ape, SAASI, and Phangorn.

For manuscripts utilizing custom algorithms or software that are central to the research but not yet described in published literature, software must be made available to editors and reviewers. We strongly encourage code deposition in a community repository (e.g. GitHub). See the Nature Portfolio guidelines for submitting code & software for further information.

Data

Policy information about availability of data

All manuscripts must include a data availability statement. This statement should provide the following information, where applicable:

- Accession codes, unique identifiers, or web links for publicly available datasets
- A description of any restrictions on data availability
- For clinical datasets or third party data, please ensure that the statement adheres to our policy

All whole genome sequencing data are publicly available in the European Nucleotide Archive (https://www.ebi.ac.uk/ena/browser/home). Strain meta-data (accession number, country of isolation, estimated sampling date) are available within the paper and its Supplementary Information.

Research involving human participants, their data, or biological material

Policy information about studies with human participants or human data. See also policy information about sex, gender (identity/presentation), and sexual orientation and race, ethnicity and racism.

Reporting on sex and gender	NA
Reporting on race, ethnicity, or other socially relevant groupings	NA
Population characteristics	NA
Recruitment	NA
Ethics oversight	NA

Note that full information on the approval of the study protocol must also be provided in the manuscript.

Field-specific reporting

Please select the one below that is the best fit for your research. If you are not sure, read the appropriate sections before making your selection.

☐ Life sciences ☐ Behavioural & social sciences ☒ Ecological, evolutionary & environmental sciences

For a reference copy of the document with all sections, see nature.com/documents/nr-reporting-summary-flat.pdf

Ecological, evolutionary & environmental sciences study design

All studies must disclose on these points even when the disclosure is negative.

Study description	We searched a large publicly available dataset of ~200,000 Mtb whole genome sequences to identify strains related to a highly successful lineage 4.2/Ural MDR strain circulating in Moldova. We report the inferred country of origin, the relative rates of migration among countries, and the relative fitness of this clade.
Research sample	We include 5909 lineage 4.2 M. tuberculosis genomes. We identify 1604 strains that are similar to the rapidly spreading MDR lineage previously described in Moldova.
Sampling strategy	We included all genomes meeting our criteria (i.e. no sampling). We queried the European Nucleotide Archive (ENA) on 18 February 2024 for all M. tuberculosis genomes (n = 196,547 accessions). We identified 7165 unique sample accessions that were profiled as Mtb lineage 4.2. After quality control checks, we identifited 5909 Mtb lineage 4.2 sequences.
Data collection	We collected data from the ENA, and we cross-reference meta-data with data from TB Profiler, where available.
Timing and spatial scale	We queried ENA on 18 February 2024. All genomes were collected between 1994 and 2023.
Data exclusions	We excluded laboratory and reference strains, other Mycobacteria species in the MTBC, and samples isolated from non-human hosts. Alignments with less than 80% mapping to the H37Rv reference strain and an average read depth below 50x were removed, along with any sample with evidence of mixed infection. In cases where samples had multiple run accessions (duplicate or re-sequenced isolates), alignments with the highest mapping and average read depth were retained.
Reproducibility	The ENA search is reproducible using code that are publicly available on github: https://github.com/bensobkowiak/ENA_search. In the methods section, we specify which software packages and settings were used to produce analytic results.
Randomization	This is an observational study (i.e. no randomization). Observations were grouped based upon either their reported country of origin or their phylogenetic relationships.
Blinding	This is an observational study. We did not use blinding during data acquisition or analysis.

Did the study involve field work? ☐ Yes ☒ No

Reporting for specific materials, systems and methods

We require information from authors about some types of materials, experimental systems and methods used in many studies. Here, indicate whether each material, system or method listed is relevant to your study. If you are not sure if a list item applies to your research, read the appropriate section before selecting a response.

Materials & experimental systems

n/a	Involved in the study
☒	☐ Antibodies
☒	☐ Eukaryotic cell lines
☒	☐ Palaeontology and archaeology
☒	☐ Animals and other organisms
☒	☐ Clinical data
☒	☐ Dual use research of concern
☒	☐ Plants

Methods

n/a	Involved in the study
☒	☐ ChIP-seq
☒	☐ Flow cytometry
☒	☐ MRI-based neuroimaging

Plants

Seed stocks

Report on the source of all seed stocks or other plant material used. If applicable, state the seed stock centre and catalogue number. If plant specimens were collected from the field, describe the collection location, date and sampling procedures.

Novel plant genotypes

Describe the methods by which all novel plant genotypes were produced. This includes those generated by transgenic approaches, gene editing, chemical/radiation-based mutagenesis and hybridization. For transgenic lines, describe the transformation method, the number of independent lines analyzed and the generation upon which experiments were performed. For gene-edited lines, describe the editor used, the endogenous sequence targeted for editing, the targeting guide RNA sequence (if applicable) and how the editor was applied.

Authentication

Describe any authentication procedures for each seed stock used or novel genotype generated. Describe any experiments used to assess the effect of a mutation and, where applicable, how potential secondary effects (e.g. second site T-DNA insertions, mosiacism, off-target gene editing) were examined.
